# Incidence and prevalence of headache in influenza: A 2010–2021 surveillance‐based study

**DOI:** 10.1111/ene.16349

**Published:** 2024-05-21

**Authors:** David García‐Azorín, Laura Santana‐López, Ana Ordax‐Díez, José Eugenio Lozano‐Alonso, Diego Macias Saint‐Gerons, Yésica González‐Osorio, Silvia Rojo‐Rello, José M Eiros, Javier Sánchez‐Martínez, Álvaro Sierra‐Mencía, Andrea Recio‐García, Ángel Luis Guerrero‐Peral, Ivan Sanz‐Muñoz

**Affiliations:** ^1^ Department of Medicine, Faculty of Medicine Universidad de Valladolid Valladolid Spain; ^2^ Headache Unit, Department of Neurology Hospital Clínico Universitario de Valladolid Valladolid Spain; ^3^ Dirección General de Salud Pública e Investigación Desarrollo e Innovación, Gerencia Regional de Salud, Junta de Castilla y Leon Valladolid Spain; ^4^ Department of Medicine University of Valencia, Instituto de Investigación Sanitaria de Valencia (INCLIVA) Health Research Institute and Centro de Investigación Biomédica en Red de Salud Mental (CIBERSAM) Valencia Spain; ^5^ Department of Microbiology Hospital Clínico Universitario de Valladolid Valladolid Spain; ^6^ National Influenza Center Valladolid Spain; ^7^ Fundación Instituto de Estudios de Ciencias de la Salud de Castilla y Leon, Instituto de Ciencias de la Salud de Castilla y Leon (ICSCYL) Soria Spain

**Keywords:** epidemiology, headache disorders, infections, migraine, virus diseases

## Abstract

**Background and purpose:**

Influenza is a common cause of acute respiratory infection, with headache being one of the symptoms included in the European Commission case definition. The prevalence of headache as a symptom of influenza remains unknown. We aimed to describe the incidence and prevalence of headache in patients with influenza.

**Methods:**

All consecutive patients who met the definition criteria of influenza‐like illness during the influenza seasons 2010–2011 through 2021–2022 were included. The seasonal cumulative incidence of influenza per 1000 patients at risk and the prevalence of headache as an influenza symptom were calculated, including the 95% confidence intervals (CIs). Subgroup analyses were done based on patients' sex, age group, microbiological confirmation, vaccination status, and influenza type/subtype/lineage.

**Results:**

During the study period, 8171 patients were eligible. The incidence of headache in the context of influenza varied between 0.24 cases per 1000 patients (season 2020–2021) and 21.69 cases per 1000 patients (season 2017–2018). The prevalence of headache was 66.1% (95% CI = 65.1%–67.1%), varying between 49.6% (season 2021–2022) and 80.1% (season 2010–2011). The prevalence of headache was higher in women (67.9% vs. 65.7%, *p* = 0.03) and higher in patients between 15 and 65 years old. Headache was more prevalent in patients infected with B subtypes than A subtypes (68.7% vs. 56.9%, *p* < 0.001). There were no notable differences regarding vaccination status or microbiological confirmation of the infection.

**Conclusions:**

Headache is a common symptom in patients with influenza, with a prevalence higher than that observed in other viral infections.

## INTRODUCTION

Influenza is the clinical syndrome caused by an infection with influenza viruses. Respiratory tract infections are the second global cause of disability‐adjusted life‐years [1]. The influenza virus is responsible for 11.5% of lower respiratory tract infections [2]. The diagnosis of influenza can be based on microbiological detection by polymerase chain reaction from a respiratory tract sample or based on clinical presentation. The World Health Organization criteria for influenza‐like illness requires an acute respiratory illness (ARI) with onset during the past 7 days, with at least one of the following general symptoms: fever or low‐grade fever, malaise, headache, and/or myalgia; and at least one of the following three respiratory symptoms: cough, odynophagia, and/or dyspnea [3].

Headache is a frequent symptom in systemic viral infections. It has been recently studied in patients with coronavirus disease 2019 (COVID‐19), with a frequency that varies between 10% and 75% depending on the studies, but in the range of 20%–25% in the best designed studies and in meta‐analyses [4, 5]. In the case of influenza, current evidence relies on two small studies, which included 37 and 279 patients and reported the prevalence of headache to be 32.4% and 60%, respectively [6, 7].

In the present study, we aimed to describe the incidence and prevalence of headache as a symptom of influenza. As secondary objectives, the prevalence of headache as an influenza symptom was analyzed depending on the patients' sex, age, and vaccination status and the circulating strain of influenza.

## METHODS

### Study design

The influenCEF study is an observational descriptive study that aims to characterize the frequency, phenotype, risk factors, duration, and pathophysiology of headache as a symptom of influenza infection. Herein, we report the data from the epidemiological part of the study with a cross‐sectional study design. The study protocol was registered in ClinicalTrials.gov (NCT05704335). The study was conducted and reported in accordance with the Strengthening the Reporting of Observational Studies in Epidemiology criteria [8]. The East Valladolid Ethics Review Board approved the study (PI 22–2884).

### Study setting

The study was done in Castile and Leon, a region localized in the northwest of Spain. The study was done in collaboration with the Healthcare Sentinel Network of Castile and Leon (*Red Centinela Sanitaria de Castilla y Leon*), the Headache Unit and the Department of Microbiology of the Hospital Clínico Universitario de Valladolid, and the National Influenza Center of Valladolid.

### Study procedures

The Health Sentinel Network of Castile and Leon has carried out active influenza surveillance from the 1996–1997 season to the 2019–2020 season within the Influenza Comprehensive Surveillance Program (*Programa de Vigilancia Integrada de la Gripe*) and from 2020–2021 onward within the Acute Respiratory Infections Comprehensive Surveillance Program (*P* *rograma de Vigilancia Integrada de las Infecciones Respiratorias Agudas* [VIGIRA]) [9]. This program is integrated into the European Influenza Surveillance Network of the European Center for Disease Prevention and Control through the ARI Surveillance System (*Sistema de Vigilancia de Infección Respiratoria Aguda*) of the Carlos III Health Institute.

The current VIGIRA program includes patients attended by general practitioners and primary care pediatricians, involved in a sentinel network in primary care settings, for an ARI.

All patients who meet the definition criteria of influenza‐like illness (ILI) [10] are registered as clinical cases. After clinical identification, the patient is sampled (nasopharyngeal swab) and diagnosed by various multiplex real‐time polymerase chain reaction (RT‐PCR) systems depending on the capabilities of the core laboratories of Castile and Leon involved in the microbiological diagnostics (Luminex NxTag Respiratory Panel‐Luminex; FilmArray Respiratory Panel 2.1. plus; GenexPert Flu/RSV Cepheid); the diagnostic panels include several viruses and bacteria, including SARS‐CoV‐2. The full details are available in Appendix S1. The sample is then sent to the National Influenza Center for confirmation and subtyping of the influenza strain by specific centers for disease control and prevention (CDC) RT‐PCR. Active monitoring is done by approximately 100 health care providers, including general practitioners, pediatricians, and nurses, covering a population that, in the current study, ranged between 27,074 (season 2015–2016) and 58,299 (season 2021–2022) [11].

### Study population

Patients were included if they fulfilled the ILI criteria [3]: (i) acute respiratory illness with onset during the past 7 days; (ii) presence of at least one of the following symptoms: fever or low‐grade fever, malaise, headache, or myalgia; and (iii) presence of at least one of the following respiratory symptoms: cough, odynophagia, or dyspnea.

Patients were excluded if the diagnosis was better accounted for by another virus and/or bacteria.

### Study period

Twelve consecutive surveillance seasons were considered, beginning with the 2010–2011 season and ending with the 2021–2022 season. An influenza surveillance season is defined as the calendar weeks during which influenza is most likely to circulate at detectable levels (as per sentinel surveillance systems), is based on historical knowledge, and usually corresponds to the period between the 40th week of the year and the 20th week of the year after the World Health Organization Europe [3].

### Study variables

A series of variables were consistently collected, including the season, the type of diagnosis (clinical vs. microbiologically confirmed), sex at birth, age at the moment of the infection (stratified into the following age groups: 0–4, 5–14, 15–44, 45–64, 65–74, and 75 years or older), influenza strain, and vaccination status. Other variables that were consistently collected were not assessed in this study, but these are listed in Appendix S1.

### Bias

Selection bias was unlikely due to the inclusion of all consecutive patients, at a population‐level, throughout 12 influenza seasons. Outcome bias was also deemed unlikely, because all patients were systematically and consistently evaluated by the VIGIRA health care providers. Because headache is an early symptom in acute systemic infections [2, 4, 6, 7], the evaluation was likely completed after a sufficient amount of time for the headache outcome to appear, but in some patients with a more delayed onset, the true headache prevalence may be underestimated. Memory bias was avoided by the evaluation of patients during the active period of the disease, while the symptoms were still present. Prior history of headache was not assessed within the study questionnaire, and therefore, the true prevalence of headache attributed to infection with influenza may be overestimated; however, according to the International Classification of Headache Disorders, a twofold increase in the frequency and/or intensity of a preexisting headache is the criterion to diagnose a secondary headache in a patient with a prior primary headache disorder [11].

### Statistical analysis

Qualitative and ordinal variables are described as frequency and percentage and quantitative variables as mean and standard deviation (SD) or median and interquartile range (IQR). To calculate the seasonal cumulative incidence of influenza, the number of incident (new) cases per 1000 inhabitants at risk was estimated every influenza season, over the total number of patients actively monitored by the VIGIRA network within each specific season [12], including the 95% confidence interval (CI). CIs were calculated according to the Newcombe and Wilson methods [13, 14]. The prevalence of headache was calculated as the proportion of patients who reported headache during the course of the influenza disease out of the total number of patients diagnosed with influenza infection, including the 95% CI [13, 14]. Subgroup analyses were done based on patients' sex, age group, microbiological confirmation, vaccination status, and influenza type/subtype/lineage. To manage missing data, two analyses were conducted, one per intention‐to‐treat (ITT), which included all patients and assumed that patients with missing data did not have headache; and another per‐protocol (PP) analysis, which included only patients with valid data in the denominator.

For hypothesis testing between two groups, chi‐squared tests and Fisher exact tests were carried out. Statistical differences were considered significant if the *p* value was <0.05. Univariate and multivariable logistic regression models were built, including “headache” as the dependent variable, to evaluate the strength of the association with influenza. In the regression analysis, odds ratios (ORs) with their corresponding 95% CIs were estimated for all covariates using the backward strategy. Multicollinearity was assessed by the variance inflation factor (VIF) and was considered critical when VIF was >5.

## RESULTS

During the study period, 8171 patients fulfilled the eligibility criteria. There was a lack of data on headache presence ascertainment in 82 of 8171 (1.0%) patients, including three patients from the 2020–2021 season and 78 patients from the 2021–2022 season.

### Incidence of influenza

Table 1 shows the age‐adjusted incidence of influenza between the seasons 2010–2011 and 2021–2022, which was lowest in the 2020–2021 season and highest in the 2019–2020 season.

**TABLE 1:** Incidence of influenza per 1000 patients at risk, stratified by patient age groups.

Season	Age							
	0–4 years	5–14 years	15–24 years	25–44 years	45–64 years	65–74 years	75+ years	All ages
2010–2011	32.39 (25.77–40.59)	36.81 (31.61–42.81)	25.6 (20.04–32.59)	18.68 (15.92–21.88)	9.60 (7.55–12.19)	4.86 (2.70–8.53)	2.41 (1.23–4.59)	17.48 (16.09–19.00)
2011–2012	42.35 (34–68‐51.58)	34.72 (29.59–40.68)	19.66 (14.9–25.83)	19.63 (16.83–22.89)	16.42 (13.77–19.54)	9.52 (6.35–14.12)	6.13 (4.13–9.04)	19.83 (18.36–21.41)
2012–2013	58.58 (47.37–72.16)	58.62 (51.25–66.97)	15.87 (11.50–21.76)	14.35 (11.88–17.30)	14.84 (12.32–17.86)	10.48 (7.06–15.43)	6.45 (4.34–9.51)	20.62 (19.06–22.32)
2013–2014	46.74 (37.51–58.03)	29.35 (24.43–35.20)	23.47 (17.98–30.52)	14.05 (11.50–17.14)	13.25 (10.84–16.16)	10.87 (7.32–16.01)	5.82 (3.78–8.86)	17.27 (15.82–18.84)
2014–2015	97.66 (84.77–112.21)	83.16 (75.18–91.89)	25.44 (19.75–32.63)	19.73 (16.60–23.41)	14.93 (12.40–17.95)	14.68 (10.55–20.31)	3.51 (1.95–6.16)	30.71 (28.78–32.28)
2015–2016	99.31 (85.87–114.55)	48.84 (42.51–56.04)	12.07 (8.19–17.65)	15.24 (12.37–18.74)	12.15 (9.82–15.0)	9.65 (6.33–14.56)	4.29 (2.49–7.24)	22.54 (20.83–24.38)
2016–2017	82.02 (69.43–96.59)	38.42 (32.84–44.86)	26.48 (20.53–34.04)	16.85 (13.93–20.35)	20.53 (17.50–24.06)	17.54 (12.82–23.86)	16.77 (12.67–22.11)	25.81 (23.99–27.77)
2017–2018	89.98 (79.93–101.11)	61.75 (56.28–67.72)	22.78 (17.57–29.42)	17.37 (14.54–20.73)	19.2 (16.47–22.36)	21.53 (16.75–27.60)	10.27 (7.38–14.22)	32.67 (30.84–34.59)
2018–2019	92.89 (82.90–103.93)	59.06 (53.74–64.87)	18.82 (14.15–24.94)	19.48 (16.38–23.13)	14.99 (12.59–17.83)	15.22 (11.21–20.57)	9.53 (6.71–13.45)	31.43 (29.63–33.33)
2019–2020	101.22 (91.11–112.23)	62.5 (57.05–68.43)	17.52 (12.67–24.16)	17.33 (10.94–16.33)	11.91 (9.61–14.72)	6.2 (3.67–10.28)	4.1 (2.28–7.20)	32.73 (30.81–34.77)
2020–2021	0.88 (0.28–2.24)	0.65 (0.29–1.41)	0.29 (0.15–1.89)	0.78 (0.34–1.69)	0.33 (0.11–0.91)	0 (0–1.06)	0 (0–0.90)	0.46 (0.30–0.71)
2021–2022	15.83 (12.77–19.58)	6.03 (4.81–7.56)	7.22 (4.93–10.49)	5.25 (4.0–6.86)	3.49 (2.61–4.65)	1.74 (0.85–3.43)	1.18 (0.52–2.55)	5.41 (4.84–6.05)

*Note*: Values are presented as incidence (95% confidence interval).

### Incidence of headache in the context of influenza

Table 2 shows the age‐specific incidence of headache in patients with Influenza. The incidence of headache ranged between 0.24 (season 2020–2021) and 21.69 (season 2017–2018). The incidence was highest in patients aged 5–14 years.

**TABLE 2:** Incidence of influenza cases with headache per 1000 patients at risk, stratified by patient age groups.

Season	Age							
	0–4 years	5–14 years	15–24 years	25–44 years	45–64 years	65–74 years	75+ years	All ages
2010–2011	20.03 (14.92–26.78)	30.64 (25.90–36.18)	21.78 (16.68–28.33)	15.42 (12.93–18.37)	7.82 (5.98–10.19)	3.74 (1.90–7.10)	2.17 (1.06–4.28)	14.01 (12.76–15.37)
2011–2012	22.9 (17.37–30.08)	28.55 (23.91–34.04)	17.80 (13.29–23.74)	16.90 (14.30–19.95)	14.13 (11.69–17.05)	7.69 (4.89–11.94)	4.32 (2.68–6.87)	15.83 (14.52–17.26)
2012–2013	38.16 (29.19–49.59)	47.69 (41.03–55.33)	13.49 (9.50–19.02)	11.40 (9.22–14.08)	11.49 (9.29–14.18)	6.99 (4.27–11.26)	4.54 (2.81–7.23)	15.90 (14.53–17.40)
2013–2014	17.89 (12.39–25.61)	23.58 (19.20–28.91)	19.36 (14.41–25.88)	10.68 (8.48–13.42)	9.67 (7.64–12.21)	8.05 (5.06–12.65)	5.06 (3.18–7.95)	12.34 (11.12–13.69)
2014–2015	36.82 (28.96–46.63)	59.53 (52.75–67.11)	21.80 (16.57–28.56)	16.32 (13.49–19.71)	11.07 (8.91–13.72)	10.04 (6.71–14.91)	3.24 (1.76–5.82)	20.83 (19.25–22.55)
2015–2016	33.29 (25.59–43.12)	38.23 (32.64–44.72)	8.62 (5.42–13.54)	11.26 (8.82–14.35)	8.06 (6.19–10.45)	7.64 (4.74–12.15)	2.29 (1.06–4.69)	14.09 (12.75–15.57)
2016–2017	27.34 (20.23–36.73)	29.91 (25.01–35.70)	20.50 (15.32–27.32)	11.64 (9.24–14.63)	14.66 (12.12–17.72)	11.69 (7.93–17.09)	9.68 (6.65–13.97)	16.65 (15.19–18.25)
2017–2018	31.37 (25.46–38.54)	48.14 (43.30–53.49)	17.08 (12.63–23.00)	13.34 (10.87–16.34)	14.48 (12.13–17.28)	13.8 (10.04–18.85)	7.03 (4.89–10.43)	21.69 (20.21–23.28)
2018–2019	26.78 (21.45–33.34)	42.29 (37.78–43.73)	15.44 (11.24–21.09)	16.38 (13.55–19.77)	11.42 (9.33–13.94)	11.76 (8.29–16.59)	7.01 (4.64–10.49)	19.92 (18.49–21.64)
2019–2020	22.15 (17.49–27.98)	41.9 (37.43–46.87)	11.70 (7.81–17.36)	12.08 (9.48–15.35)	9.61 (7.56–12.17)	5.04 (2.80–8.84)	3.15 (1.60–5.99)	17.80 (16.38–19.33)
2020–2021	0 (0–1.06)	0.37 (0.12–1.03)	0 (0–1.39)	0.56 (0.21–1.38)	0.25 (0.06–0.79)	0 (0–1.06)	0 (0–0.90)	0.24 (0.13–0.44)
2021–2022	1.46 (0.68–2.99)	2.14 (1.45–3.13)	2.99 (1.62–5.37)	2.72 (1.85–3.96)	2.28 (1.58–3.26)	0.58 (0.15–1.85)	0.51 (0.15–1.80)	1.97 (1.63–2.37)

*Note*: Values are presented as incidence (95% confidence interval).

### Global and seasonal prevalence of headache

**FIGURE 1:**
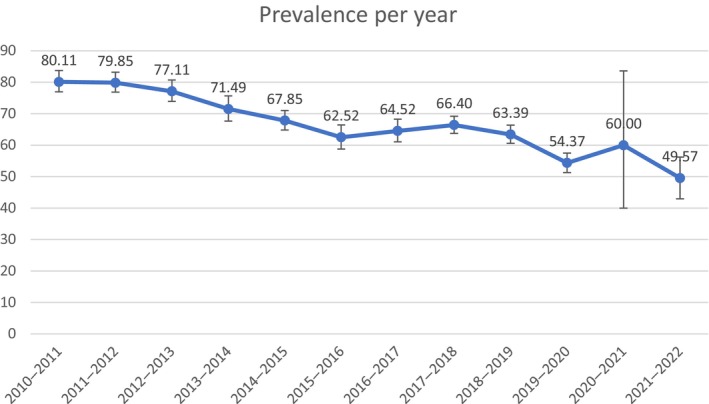
Annual prevalence of headache with 95% confidence intervals.

Headache was reported by 5402 of 8171 (66.11%, 95% CI = 65.07%–67.14%) influenza patients in the 2010–2022 period. Figure 1 shows the annual prevalence of headache. The prevalence varied between 49.6% (season 2021–2022) and 80.1% (season 2010–2011). In the two seasons where the presence of headache variable was missing for a total of 82 patients, if all missing data were headache‐positive, the true prevalence may have been as high as 75% (12/20 [60%] vs. 12 + 3 [missing] = 15/20 [75%]) for the 2020–2021 season, and as high as 83.5% (114/230 [49.6%] vs. 114 + 78 [missing] = 192/230 [83.5%]) for the 2021–2022 season.

### Prevalence of headache depending on type of diagnosis in patients with influenza

A sample was collected for microbiological confirmation in 2451 of 8171 (30.0%) cases. Samples were taken from 1444 of 2451 (58.9%) patients with headache and 1007 of 2451 (41.1%) patients without headache (*p* < 0.001). The prevalence of headache was significantly lower in patients where a sample was obtained compared to patients where a sample was not obtained (30.7% vs. 35.1%, *p* < 0.001).

Influenza infection was confirmed in 1671 patients, and data about the presence of headache was available in 1556 of 1671 (93.1%) of these cases. Headache was present in 997 of 1671 (59.7%) cases that had a positive influenza result, compared to 703 of 1053 (66.8%) cases with no microbiological confirmation (*p* < 0.001).

**FIGURE 2:**
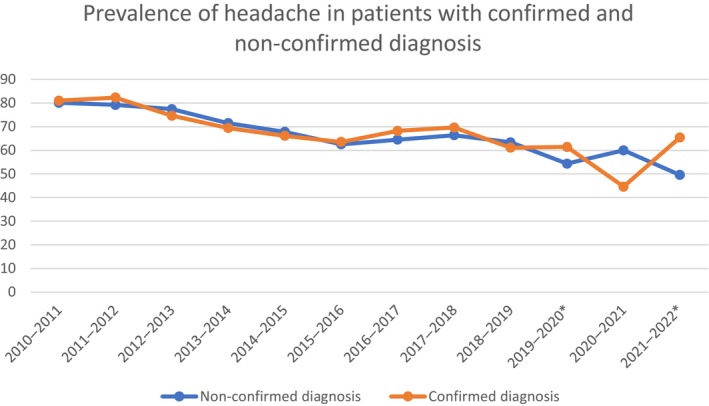
Prevalence of headache in patients with confirmed versus nonconfirmed diagnosis. *Denotes statistical significance.

Across influenza seasons, the prevalence of headache in patients with influenza infection was not different between patients with microbiologically confirmed infection those with only clinical diagnosis except for the 2019–2020 season, when it was 8.3% more frequent in patients with nonconfirmed diagnosis, and the 2021–2022 season, when it was 3.2% more frequent in patients with confirmed diagnosis (Table S1, Figure 2).

### Prevalence of headache by patients' sex

**FIGURE 3:**
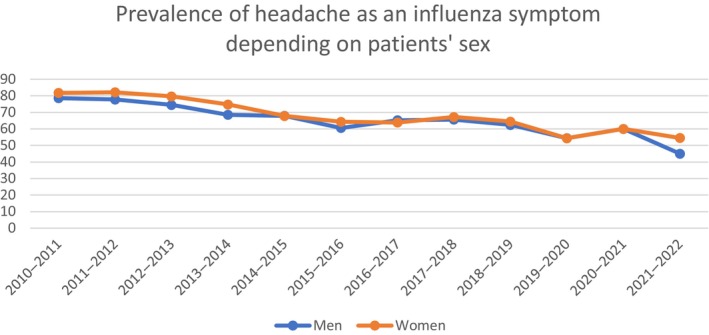
Prevalence of headache in men and women.

The overall prevalence of headache in patients with influenza infection was slightly higher in women than in men (2701/3976 [67.93%] vs. 2700/4106 [65.76%], *p* = 0.0379). There were no statistically significant differences in the prevalence of headache by influenza season when analyzed by sex (Figure 3).

### Prevalence of headache by patients' age

**FIGURE 4:**
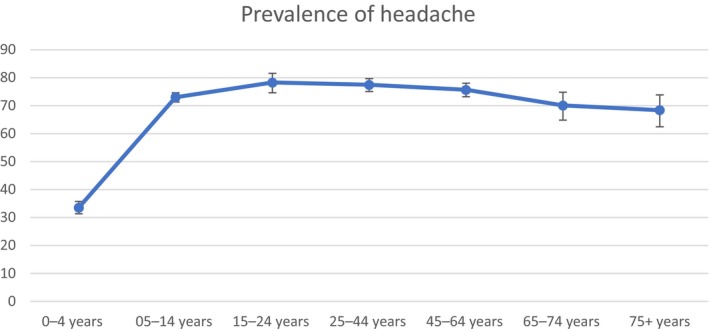
Prevalence of headache by age group with 95% confidence intervals.

There were statistically significant differences in the prevalence of headache across age groups (*p* < 0.001). The prevalence of headache ranged from 33.51% (0–4 years) to 78.26% (15–24 years). Figure 4 shows the prevalence of headache within the different age groups in the ITT analysis and PP analysis. Table S2 shows the age‐adjusted prevalence and the 95% CIs in the ITT and PP analyses.

### Prevalence of headache depending on vaccination status

Three hundred sixty‐three patients were infected after being vaccinated (4.3%). The global prevalence of headache within vaccinated, infected individuals was 65.01% (95% CI = 59.83–69.87), compared to 65.51% (95% CI = 64.46–66.55) within nonvaccinated individuals (*p* = 0.845).

### Prevalence of headache depending on influenza type/subtype/lineage

There were statistically significant differences in the prevalence of headache depending on the influenza type/subtype/lineage identified by RT‐PCR. Headache was more prevalent in patients infected with B subtypes than with A subtypes (68.73%, 95% CI = 63.81%–73.27% vs. 56.93%, 95% CI = 54.17%–59.65%, *p* < 0.001). Table 3 and Figure S1 show headache prevalence values across patients infected with the different influenza lineages for both ITT and PP analyses.

**TABLE 3:** Prevalence of headache depending on influenza lineage.

Type/subtype/lineage	Prevalence of headache, ITT, %	Prevalence of headache, PP, %
All A subtypes, *n* = 1284	56.93 (95% CI = 54.17–59.65)	62.53 (95% CI = 59.68–65.30)
AH3, *n* = 834	53.71 (95% CI = 50.26–57.14)	60.87 (95% CI = 57.23–64.40)
AH3N2, *n* = 2	100 (95% CI = 19.79–100)	100 (95% CI = 19.79–100)
AnH1N1, *n* = 377	66.05 (95% CI = 60.99–70.77)	66.94 (95% CI = 61.87–71.65)
A NS, *n* = 73	46.58 (95% CI = 34.95–58.56)	55.74 (95% CI = 42.51–68.24)
All B subtypes, *n* = 387	68.73 (95% CI = 63.81–73.27)	68.73 (95% CI = 63.81–73.27)
B NS, *n* = 187	65.78 (95% CI = 58.44–72.45)	65.78 (95% CI = 58.44–72.45)
B/Victoria, *n* = 38	78.95 (95% CI = 62.22–89.86)	78.95 (95% CI = 62.22–89.86)
B/Yamagata, *n* = 162	69.75 (95% CI = 61.75–76.58)	69.75 (95% CI = 61.75–76.58)

Abbreviations: ITT, intention‐to‐treat; NS, nonsubtyped; PP, per‐protocol.

### Variables associated with presence of headache

In the univariate logistic regression analysis, female sex, age, and influenza strain were associated with a higher probability of having headache, whereas the influenza season, whether a sample was collected, and confirmed diagnosis were associated with a lower probability of headache (Table S3). In the multivariate logistic regression analysis, age group 0–4 years (OR = 0.282, 95% CI = 0.171–0.465, *p* < 0.001), season (OR = 0.999984, 95% CI = 0.999980–0.999987, *p* < 0.001), and the influenza strain B (OR = 1.065, 95% CI = 1.006–1.127, *p* = 0.030) remained statistically significant.

## DISCUSSION

In the present study, the incidence and prevalence of headache in patients with influenza was revisited in a study of cases from a sentinel network that collected data during 12 consecutive influenza seasons. In the pre‐COVID‐19 era, the incidence was in the range of 12–22 cases of headache per 1000 patient‐years. The prevalence of headache was in the 50%–70% range and varied slightly depending on the patients' sex, age, and vaccination status and the circulating type/subtype/lineage.

The incidence of headache in patients with influenza (between 24 and 2169 per 100,000 person‐years) was higher than the age‐standardized incidence (per 100,000 people‐year) in other notable diseases, such as meningitis (39 per 100,000), encephalitis (90 per 100,000), stroke (203 per 100,000), or traumatic brain injury (369 per 100,000) [15]. The proportion of influenza patients who reported headache significantly exceeded the prevalence of primary headache disorders, which is estimated to be 26.1% for tension‐type headache and 14.4% for migraine [16]. In addition, migraine (female prevalence 17.0%, male prevalence 8.6%) and tension‐type headache (female prevalence 27.1%, male prevalence 23.4%) are more frequent in women [17]; however, in the case of headache in influenza patients, the difference in prevalence by sex was less pronounced (+3%). Even if some of the included patients in this study were suffering from an exacerbation of a primary headache disorder, the prevalence of headache observed in this study could not solely be explained by a preexisting headache history.

In 2011, the World Health Organization set novel diagnostic criteria for ILIs. ILIs are a group of disorders that include influenza, respiratory syncytial virus, rhinovirus, adenovirus, parainfluenza viruses, human metapneumovirus, and coronaviruses [18, 19]. In our setting, microbiological confirmation was routinely obtained in one third of cases, which ensured the accuracy of the clinical diagnosis [3, 18]. The differences in headache prevalence in patients with confirmed and nonconfirmed diagnosis were mild and <5% in all seasons except for the 2019–2020 season, when it was 8.3% more prevalent in patients with confirmed diagnosis.

The overall prevalence of headache in our study may be underestimated due to the significantly lower prevalence in patients aged 0–4 years, where only one third of patients reported headache. Headache is a subjective symptom, and the collaboration of the patient is needed to report its presence [11]. Therefore, the evaluation of patients with speech disorders, of patients with severe cognitive disturbances, or of very young infants may not be precise if they cannot describe whether they suffer from headache [11], and this may pose some degree of attrition bias. In patients older than 5 years, the prevalence of headache was >70% and was slightly lower in patients older than 75 years [17]. In addition, in this setting, the incidence of influenza was higher in patients aged 0–4 years, similar to other previous series [20].

Influenza is a vaccine‐preventable disease. It was remarkable that only 4.3% of the samples that tested positive for influenza were in patients already vaccinated that year. The global prevalence of influenza vaccination in Spain has been estimated to be approximately 30% in the global population, >42% in patients with at least one indication for vaccination, and >60% in patients aged >65 years [21, 22]. In these patients, the prevalence of headache was almost the same as in nonvaccinated patients. In the case of other infectious diseases that may cause headache, such as COVID‐19, one of the main pathophysiological theories involves the immune response [6, 23], and vaccination status may enhance the immune response, modifying the clinical phenotype of the disease [23, 24, 25, 26]. In contrast, the prevalence of headache did vary depending on the specific influenza strain. The lowest headache prevalence was observed in patients infected with AH3 and ANS strains; the reason for this is uncertain. In the case of COVID‐19, patients with headache have a better prognosis and a more benign course of the disease [23]. We hypothesize that in the case of influenza, with the type/subtype/lineages where hospitalization of patients is more frequent [27, 28], such as AH3, the innate immune response could be less efficient and therefore, headache could be less prevalent [25, 26].

Multiple factors may modulate the presence of headache during the course of influenza infection, including individual predisposition, the severity of the disease, vaccination status, or the specific influenza strain. A multivariate analysis was conducted to disentangle which variables may be acting as confounders, and the main drivers appeared to be patients' age, the influenza season, and the influenza type/subtype/lineage. In the present study, we did not assess the presence of other comorbid disorders, the severity of influenza, or the presence of other symptoms, which could play a role, as has been observed in the case of COVID‐19 [23, 24].

The main limitations of the study are the limited geographical and racial representativeness, the lack of systematic microbiological confirmation, and the lack of specific evaluation of the headache phenotype and prior history of headache. In a prospective cohort study that was done in 2023, in the same population, that characterized the clinical phenotype of the headache, the proportion of patients with headache attributed to influenza infection who reported prior history of headache was 26.7% [29]. According to the only population‐level study that has assessed the prevalence of migraine in Spain, prevalence was estimated to be 12.6% (95% CI = 11.6–13.6) [30]. In the case of tension‐type headache, prevalence has been estimated to be approximately 31.1% (95% CI = 22.8%–39.2%) in our region [17]. Therefore, the observed prevalence of headache in this study could not be explained solely by a worsening of a preexisting headache. In addition, in this study we were not able to evaluate whether each patient fulfilled the International Classification of Headache Disorders criteria for acute headache attributed to a systemic viral infection [11], but in a recent study conducted in the same population the proportion of patients with headache during the course of influence who fulfilled these criteria was 100% [29]. There was no follow‐up of the patients, so the delayed onset of the headache could imply an underestimation of the true prevalence too. Furthermore, this study's evaluation of the epidemiology of headache in patients with influenza was comprehensive and was explored and analyzed from multiple perspectives, providing relevant data that did not exist in the literature before.

In conclusion, the incidence of headache in patients with influenza reached almost 322 dases per 1000 patient‐years. Two thirds of patients presented headache during the course of influenza. The prevalence of headache was higher in women, patients aged 15–65 years, and patients infected with B subtypes. Neither vaccination status nor patients' sex yielded notable differences regarding the prevalence of headache.

## AUTHOR CONTRIBUTIONS

**David García‐Azorín:** Conceptualization; methodology; writing – original draft; writing – review and editing; formal analysis. **Laura Santana‐López:** Data curation; investigation; formal analysis; writing – original draft; writing – review and editing. **Ana Ordax‐Díez:** Formal analysis; data curation; writing – review and editing. **José Eugenio Lozano‐Alonso:** Methodology; formal analysis; writing – review and editing. **Diego Macias Saint‐Gerons:** Data curation; writing – review and editing; formal analysis. **Yésica González‐Osorio:** Methodology; data curation; validation; writing – review and editing. **Silvia Rojo‐Rello:** Data curation; formal analysis; writing – review and editing. **José Eiros‐Bouza:** Formal analysis; supervision; visualization. **Javier Sánchez‐Martínez:** Data curation; formal analysis; writing – review and editing. **Álvaro Sierra‐Mencía:** Data curation; formal analysis; investigation; writing – review and editing. **Andrea Recio‐García:** Data curation; formal analysis; writing – review and editing; investigation. **Ángel Luis Guerrero‐Peral:** Conceptualization; methodology; writing – review and editing. **Ivan Sanz‐Muñoz:** Conceptualization; methodology; data curation; formal analysis; writing – original draft; writing – review and editing.

## CONFLICT OF INTEREST STATEMENT

D.G.‐A. has received honoraria for lectures/presentations from AbbVie/Allergan, Eli Lilly, Teva, Lundbeck, and Novartis. D.G.‐A. has participated in clinical trials as the principal investigator for Pfizer, BioHaven, and Lundbeck. D.G.‐A. is junior editor of *The Journal of Headache and Pain*. D.G.‐A. has received honoraria from the World Health Organization as a subject matter expert. Á.L.G.‐P. has received honoraria for lectures/presentations from AbbVie/Allergan, Eli Lilly, Teva, Lundbeck, and Novartis. Á.L.G.‐P. has participated in clinical trials as the principal investigator for Eli Lilly, Teva, AbbVie, Novartis, Amgen, and Lundbeck. None of the other authors has any conflict of interest to disclose.

## ETHICS APPROVAL

The study was conducted in line with the principles of the Declaration of Helsinki. Approval was granted by the Ethics Committee of Valladolid University Hospital (PI‐GR‐22‐2840).

## CONSENT TO PARTICIPATE

Informed consent was obtained from all individual participants included in the study.

## Supporting information


Figure S1.

Appendix S1.

## Data Availability

The data that support the findings of this study are available on request from the corresponding author. The data are not publicly available due to privacy or ethical restrictions.
