# Supplementary material for: Incidence and prevalence of headache in influenza: A 2010–2021 surveillance‐based study
Source: Eur J Neurol. 2024 May 21;31(8):e16349. doi: 10.1111/ene.16349 (PMC11236060; doi:10.1111/ene.16349)
Supplement: Supplementary file 1 — Figure S1. [file ENE-31-e16349-s001.docx]

Supplementary figure 1: Prevalence of headache (with 95% confidence intervals) depending on the influenza lineage.
