# Supplementary material for: Incidence and prevalence of headache in influenza: A 2010–2021 surveillance‐based study
Source: Eur J Neurol. 2024 May 21;31(8):e16349. doi: 10.1111/ene.16349 (PMC11236060; doi:10.1111/ene.16349)
Supplement: Supplementary file 2 — Appendix S1. [file ENE-31-e16349-s002.docx]

**Incidence and prevalence of headache in Influenza: A 2010-2021 surveillance-based study**

Supplementary appendix:

*Multiplex RT-PCR systems that were used*

Luminex NxTag Respiratory Panel-Luminex:

Adenovirus, Coronavirus 229E, Coronavirus HKU1, Coronavirus NL63, Coronavirus OC43, Human Bocavirus, Human Metapneumovirus, Influenza A, Influenza A-subtype H1, Influenza A – subtype H3, Influenza B, Parainfluenza 1, Parainfluenza 2, Parainfluenza 3, Parainfluenza 4, Respiratory Syncytial Virus A, Respiratory Syncytial Virus B, Rhinovirus/Enterovirus, SARS-CoV-2, Chlamydophila pneumoniae, Mycoplasma pneumoniae, Legionella pneumoniae.

FilmArray Respiratory Panel 2.1. plus;

Adenovirus, Coronavirus 229E, Coronavirus HKU1, Coronavirus NL63, Coronavirus OC43, SARS-CoV-2, Human metapneumovirus, Human rhinovirus/enterovirus, Influenza A virus, Influenza A virus A/H1, Influenza A virus A/H3, Influenza A virus A/H1-2009, Influenza B virus, Parainfluenza virus 1, Parainfluenza virus 2, Paralinfluenza virus 3, Parainfluenza virus 4, Respiratory syncytial virus, Bordetella parapertussis, Bordetella pertussis, Chlamydia pneumoniae, Mycoplasma pneumoniae.

GenexPert Flu/RSV Cepheid:

Influenza A virus, Influenza B virus, Respiratory Syncytial virus.

*Study variables*

A series of variables are consistently collected by the VIGIRA system, including the season, a series of demographic variables, prior medical history, clinical variables, microbiological variables, and consequences of the disease. No additional variables were obtained. Demographic variables included sex at birth, age at the moment of the infection (stratified into the following age groups: 0-4, 5-14, 15-44, 45-64, 65-74 and 75 or older), and vaccination status at the moment of the infection. Variables related to the prior medical history included prior history of cardiovascular disorders, diabetes mellitus, chronic obstructive pulmonary disease (COPD), prior history of cancer, chronic kidney disease, chronic hepatic disease, or degree three obesity (body mass index >40). Clinical variables assessed the onset of the disease within 48 hours, the sudden onset of the symptoms, the epidemiological contact with another person infected by Influenza, and the presence of fever, shivering, asthenia, myalgia, cough, dyspnea, nasopharyngeal erythema, headache, and gastrointestinal symptoms. Microbiological variables included the oropharyngeal swab test obtention, the result of the test, and the influenza subtype and lineage. Last, the consequences of the disease were assessed in terms of need of hospital referral; the medical leave, in the case of adult patients; work absenteeism, in the case of children, adolescent and youth patients.

Supplementary table 1. Prevalence of headache in influenza patients with confirmed and non-confirmed diagnosis through laboratory tests

| **Season** | **Non-laboratory confirmed diagnosis (%)** | **Laboratory confirmed diagnosis (%)** | **P value** |
| --- | --- | --- | --- |
| **2010-2011** | 80.10 (76.50-83.29) | 81.03 (68.19-89.71) | 0.999* |
| **2011-2012** | 79.23 (76.51-82.82) | 82.31 (74.42-88.23) | 0.521 |
| **2012-2013** | 77.45 (73.55-80.33) | 74.67 (63.08-83.70) | 0.639 |
| **2013-2014** | 71.48 (67.29-75.34) | 69.41 (58.34-78.70) | 0.698 |
| **2014-2015** | 67.84 (64.68-70.86) | 66.13 (57.01-74.23) | 0.698 |
| **2015-2016** | 62.52 (58.56-66.32) | 63.53 (52.32-73.50) | 0.862 |
| **2016-2017** | 64.52 (60.86-68.01) | 68.24 (57.13-77.68) | 0.497 |
| **2017-2018** | 66.40 (63.57-69.12) | 69.65 (62.72-75.82) | 0.368 |
| **2018-2019** | 63.39 (60.45-66.25) | 61.11 (53.92-67.87) | 0.543 |
| **2019-2020** | 54.37 (51.27-57.44) | 61.44 (53.20-69.09) | 0.0058 |
| **2020-2021** | 60.0 (36.41-80.02) | 44.63 (37.23-52.27) | 0.238* |
| **2021-2022** | 49.56 (42.95-56.19) | 65.43 (62.83-67.94) | 0.0001 |

- * Two-tailed Fisher exact test.

Supplementary table 2: Prevalence of headache in the intention-to-treat analysis (ITT) and per protocol analysis (PP).

| Age group | Prevalence of headache (ITT) (%) | Prevalence of headache (PP) (%) |
| --- | --- | --- |
| 00-04 years | 33.51 (95%CI: 31.38-35.72) | 34.32 (95%CI: 32.15-36.57) |
| 05-14 years | 73.00 (95%CI: 71.32-74.62) | 74.10 (95%CI: 72.42-75.71) |
| 15-24 years | 78.26 (95%CI: 74.62-81.52) | 80.21 (95%CI: 76.62-83.38) |
| 25-44 years | 77.45 (95%CI: 75.08-79.66) | 78.39 (95%CI: 76.05-80.58) |
| 45-64 years | 75.66 (95%CI: 73.16-78.00) | 76.52 (95%CI: 74.03-78.84) |
| 65-74 years | 70.06 (95%CI: 64.87-74.79) | 70.26 (95%CI: 65.07-74.99) |
| 75+ years | 68.40 (95%CI: 62.43-73.84) | 69.17 (95%CI: 63.19-74.59) |

ITT: Intention-to-treat; PP: Per protocol.

Supplementary table 3: Univariate regression analysis of the variables that were associated with a higher/lower probability of headache.

| **Variable** | **OR (95%CI)** | **P value** |
| --- | --- | --- |
| Season | 0.999987 (0.999985-0.999988) | P<0.001 |
| Sex | 1.116 (1.021-1.221) | 0.016 |
| Age 00-04 years | 0.171 (0.153-0.191) | <0.001 |
| Age 05-14 years | 1.739 (1.571-1.925) | <0.001 |
| Age 15-24 years | 1.970 (1.598-2.430) | <0.001 |
| Age 25-44 years | 1.962 (1.706-2.257) | <0.001 |
| Age 45-64 years | 1.712 (1.489-1.970) | <0.001 |
| Age 65-74 years | 1.242 (0.975-1.581) | 0.079 |
| Age 75+ years | 1.196 (0.913-1.566) | 0.193 |
| Sample collection | 0.819 (0.745-0.901) | <0.001 |
| Confirmed infection | 0.745 (0.635-0.874) | <0.001 |
| Type/subtype/lineage | 1.132 (1.077-1.190) | <0.001 |
| Vaccination status | 0.978 (0.785-1.220) | 0.845 |

All VIF values were below 1.031.
